# Spatial and temporal investigations of reported movements, births and deaths of cattle and pigs in Sweden

**DOI:** 10.1186/1751-0147-51-37

**Published:** 2009-10-07

**Authors:** Maria Nöremark, Nina Håkansson, Tom Lindström, Uno Wennergren, Susanna Sternberg Lewerin

**Affiliations:** 1 SVA, National Veterinary Institute, Department of Disease Control and Epidemiology, 75189 Uppsala, Sweden; 2 Department of Clinical Sciences, Swedish University of Agricultural Sciences, Box 7054, 750 09 Uppsala, Sweden; 3 Research Centre of Systems Biology, Ecological modelling, University of Skövde, 54128 Skövde, Sweden; 4 IFM, Theory and Modelling, Linköpings Universitet, Linköping, Sweden

## Abstract

**Background:**

Livestock movements can affect the spread and control of contagious diseases and new data recording systems enable analysis of these movements. The results can be used for contingency planning, modelling of disease spread and design of disease control programs.

**Methods:**

Data on the Swedish cattle and pig populations during the period July 2005 until June 2006 were obtained from databases held by the Swedish Board of Agriculture. Movements of cattle and pigs were investigated from geographical and temporal perspectives, births and deaths of cattle were investigated from a temporal perspective and the geographical distribution of holdings was also investigated.

**Results:**

Most movements of cattle and pigs were to holdings within 100 km, but movements up to 1200 km occurred. Consequently, the majority of movements occurred within the same county or to adjacent counties. Approximately 54% of the cattle holdings and 45% of the pig holdings did not purchase any live animals. Seasonal variations in births and deaths of cattle were identified, with peaks in spring. Cattle movements peaked in spring and autumn. The maximum number of holdings within a 3 km radius of one holding was 45 for cattle and 23 for pigs, with large variations among counties. Missing data and reporting bias (digit preference) were detected in the data.

**Conclusion:**

The databases are valuable tools in contact tracing. However since movements can be reported up to a week after the event and some data are missing they cannot replace other methods in the acute phase of an outbreak. We identified long distance transports of cattle and pigs, and these findings support an implementation of a total standstill in the country in the case of an outbreak of foot-and-mouth disease. The databases contain valuable information and improvements in data quality would make them even more useful.

## Background

There are several reasons to study movements of livestock and population dynamics; animal welfare, finances, environmental aspects and spread and control of infectious diseases. Irrespective of the disease - endemic, exotic or zoonotic - live animal movements are almost always an important route of disease spread. [1,2]

In case of an outbreak of a highly contagious animal disease such as foot-and-mouth disease (FMD), movements of livestock can be totally banned, in accordance with EU-regulations, to prevent further spread [3]. Such a standstill can include the whole country or only parts of the country. In a later stage of the outbreak regionalisation can be introduced, allowing certain activities and movements within but not between the regions. Standstills and restrictions generate great losses to the industry, while the spread of disease can induce far greater losses [4]. A better understanding of the movements of livestock could help determine standstills in the acute phase of an outbreak and to capture appropriate regions when regionalising the country. Another part in disease eradication is the implementation of protection and surveillance zones around the infected holdings. The minimum radii of these zones are 3 km and 10 km and the geographical clustering of holdings will greatly affect the number of holdings within these zones [3,5].

An improved knowledge of the movement dynamics could also increase the understanding of the spread of endemic diseases and be useful when designing control programmes for these diseases. Furthermore, knowledge of the population dynamics related to deaths could be part of early warning systems; if reported deaths were surveyed continuously an unexpected rise could indicate ongoing problems in the population [6].

Moreover, modelling is increasingly used to estimate the probability of disease spread [7]. Since the movement of animals is one (among many) factor affecting the outcome of an outbreak, movements of livestock can be used as input parameters in these models [2]. Another aspect is that data from earlier outbreaks could be used in these models to estimate the risk of spread between holdings. When it comes to rare diseases, outbreak data from other countries might be used and since the demographics of animals can differ between countries, the country-specific patterns need to be taken into account [8]. An example can be the FMD outbreak in UK in 2001 where disease initially spread through live animals markets [9,10]; such spread could not be expected in countries without live animal markets.

Within the European Union (EU) some major disease outbreaks, such as Bovine Spongiform Encephalopathy in the UK and Classical Swine Fever in Belgium and the Netherlands, resulted in improved traceability of animal movements [11-13]. Member states of the EU must keep databases including all holdings and register movements of cattle and pigs. These databases enable analyses of livestock movements and in recent years several papers on different aspects of these movements have been published [14-19]. Ideally the databases on livestock movements could be used to trace contacts and identify potentially infected holdings during outbreaks and for all the purposes mentioned above. However, it is important to validate and to assess the quality of the data [16,17,20].

The aim of this study was to investigate geographical and temporal aspects of the Swedish cattle and pig populations, in particular; 1) reported movements of pigs and cattle 2) reported births and deaths of cattle 3) geographical location of holdings. Movements to slaughter were not included in this study due to lack of detailed data.

## Methods

### Study population

**Figure 1:**
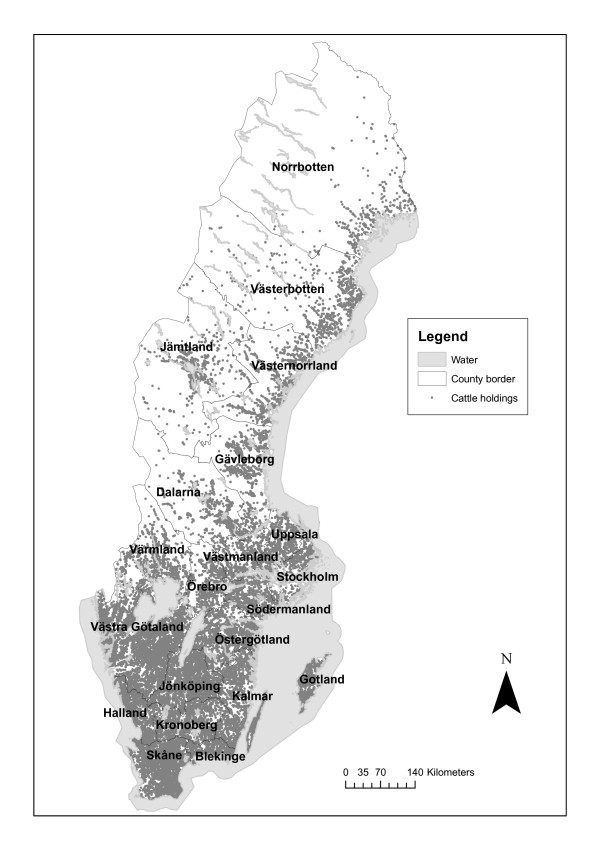
**Cattle holdings in Sweden**. Map over Sweden showing active, registered cattle holdings. © Lantmäteriet Gävle 2009. Medgivande I 2009/0830.

**Figure 2:**
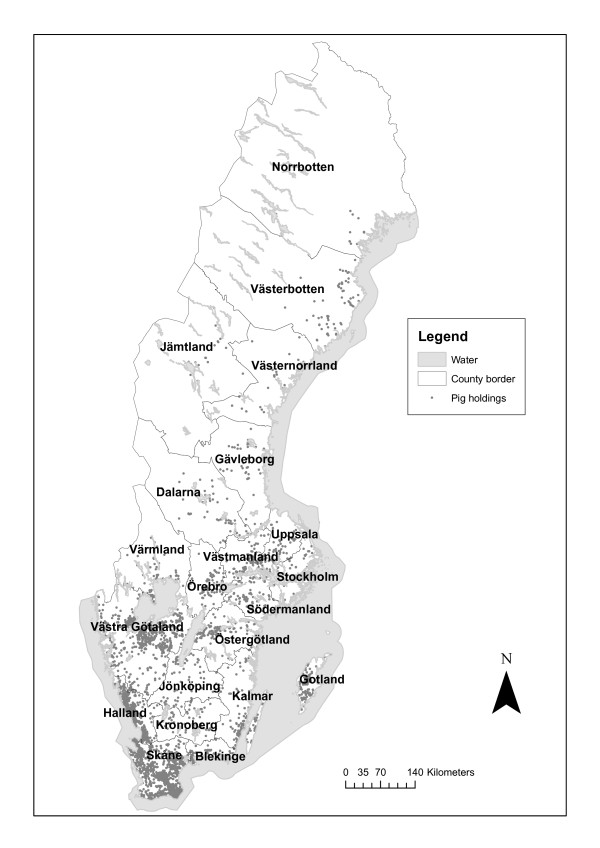
**Pig holdings in Sweden**. Map over Sweden showing active, registered pig holdings. © Lantmäteriet Gävle 2009. Medgivande I 2009/0830.

The Swedish cattle and pig populations are concentrated to the southern parts of the country (Figure 1, Figure 2). The trends for both cattle and pigs are towards increasing herdsizes and a decreasing number of herds [21]. In 2006 the number of agricultural companies with cattle was 25054 and the average herd size was 64 cattle per herd. The number of agricultural companies with pigs was 2414 and the average herd size was 116 sows and boars, and 495 piglets and pigs for fattening [22]. Auctions with live animals are rare, most animals are moved directly from one holding to another and these transactions are often mediated by the meat industry or through direct contact between farmers [23].

### Databases

The Swedish Board of Agriculture keeps databases with data on holdings and movements of cattle and pigs. All holdings where cattle or pigs are kept should be registered with a unique holding number (PPN). Holdings that are geographically separated should have different PPN and this applies also to pastures that are separated from holdings where the animals are kept. The holding database contains information on postal address, species kept and approximate number of animals kept on the holding. When animals are no longer kept on the holding this should be reported. However, this is not always done and the database contains holdings where no animals are kept but which are not registered as currently inactive.

Cattle have unique identification numbers and farmers should report the following events on individual level; birth, sale, purchase, export, import, temporarily away from holding and return after temporarily being away (for example pasture, or show), sent to slaughterhouse, slaughter on the farm, death. In addition, the date of the event should be reported. For purchases the holding of origin should be reported, and for sales the holding of destination should be reported. Reporting should be done within seven days from the events either electronically or by using a form sent by ordinary mail which is scanned into the database.

The pig database contains information on the type of production and the geographical coordinates of the holding. Pig movements are reported on group level; the holding of origin and the holding of destination, the number of pigs and date of the movement. The movements are reported only by the farmer at the PPN of destination (single reporting), within seven days after the event, either electronically or using a form sent by ordinary mail.

### Extracts from the databases for this study

The following data were obtained for the study: PPN for all holdings, postal address, species registered on the holding, geographical coordinates for pig holdings, and for cattle holdings approximate geographical coordinates obtained from a register on land use (not available for all holdings). Due to technical problems these data were obtained twice; in 2006 and in 2008. For cattle, all reported individual movements for the 12-month period from the first of July 2005 until last of June 2006 as well as reported births and deaths during the period were obtained. Moreover data on the location of individual cattle on the first and last day of this period were obtained. For pigs, group movement data, between first of July 2005 until last of June 2006 were provided. When the data for this study were collected the databases were financed by the farmers, through a fee paid for each reported movement.

### Data editing

Since both sales and purchases of cattle are reported, these movements should consist of two identical reports; one reported by the farmer at the holding of origin and one by the farmer at the holding of destination. This is in the ideal case but not always the reality.

All reported sales and purchases of cattle were matched, and for the reports without a match we tried to identify reasonable matches through a stepwise process. Reports that were identical except for different dates were kept. Reports of sales or purchases of cattle where the seller or buyer reported the wrong PPN of the holding of origin or wrong PPN for the holding of destination were kept if they could be matched on all other information and if the dates were less than one week apart. Where different types of movement of the same animal were reported (e.g. the animal reported to be sold to two different holdings), the reports that did not have a match were deleted. The reports that violated any of the following assumptions were deleted; 1) the same animal cannot leave the same holding twice without returning in between, or 2) cannot enter the same holding twice without leaving. The remaining reports were checked for consistency; if the location of the animal in the beginning and the end of the period wasn't contradicted and if the animal had been moved in a logical order without gaps between the holdings (A->B->C->D) the report was kept (Table 1).

**Table 1:** Results of data editing of reported sales and purchases of cattle during the period between July 1^st ^2005 and June 30^th ^2006 in a dataset extracted from the Swedish database of cattle movements

	**n**	**Kept in dataset**	**Excluded from dataset**
Total number of reports (sales and purchases)	515572		
Reports with perfect match	414246	x	
Reports with different dates	71158	x	
Non-matching holding reported by one of the farmers	7 216	x	
Non-matching holding reported by one of the farmers, different dates reported but less than 7 days between reported dates	2 918	x	
Reports without match, but where the location of the animal was correct according to the report	14 434	x	
Reports also reported as slaughter or to or from pasture	393		x
Duplicates and triplicates	709		x
Inconsistent transport (e.g. animal reported to have left the same holding twice without returning in between)	1 532		x
Remaining, unexplainable reports	2 975		x

Other events, such as births or deaths of cattle and movement of pigs, are single reports, and no information of pigs is registered at individual level. Consequently, the pig movement reports could not be matched or checked with location of individuals and therefore no editing of data of pig movements was done.

In the analyses we only included active holdings that had; reported at least one event or movement on or off the holding, including movements to slaughter during the period (even though these reports were not included in the further analyses) or where cattle were reported to be present on the holding at the start or at the end of the period, hereafter referred to as "holdings". In the geographical analysis, and analyses of distances of movements, only PPN with coordinates or approximate coordinates were included.

### Analyses

The number of holdings within 3 km and 10 km radius, minimum size of protection and surveillance zones [3,5] were calculated for each holding. Furthermore, holdings were investigated related to the number of other holdings they purchased animals from or sold animals to during the period.

The movements of cattle and pigs were analysed from a temporal perspective; in relation to day of the week and the week of the year (national holidays were treated as Sundays). The distance between the holding of origin and holding of destination for each movement was calculated. Further we analysed how the animals were moved within and between different counties and different regions. All animals transported between the same two holdings on the same day were regarded as one movement.

Reported births and deaths of cattle were investigated from a temporal perspective; week of the year, day of the week and day of the month, with the purpose to examine if there were weekly or seasonal trends and if there seemed to be digit preference in the reporting. Digit preference is a well known phenomenon; people tend to report certain numbers more often than others [24-27].

Day of the week and day of the month are not independent, neither are they independent from week of the year (they occur in sequence and in a year the frequency of the combinations differ among them). To investigate if reported births and deaths were equally distributed among the days we used chi-square tests, with expected value assuming equal distribution of births and deaths among the days. First, we analysed reported births and deaths in relation to the day of the week and found dependence. Secondly, we analysed reported deaths by day of the month, while adjusting for the day of the week through splitting the data and performing seven separate chi-square tests, one for each day of the week. For the reported births, the distribution over the year showed a large seasonal fluctuation which was likely to have more effect than the day of the week. Thus, for the reported births we tested the effect of day of the month with one chi-square test without adjusting for day of the week.

### Software and programming language

Analyses were performed using Perl v5.8.7.  and Matlab 7.5.0 and Matlab 7.7.0 (The MathWorks, Inc. Natick, Massachusetts, USA). The maps were generated using ArcGIS (ESRI Co., Redlands, California, USA).

## Results and Discussion

### Movements

**Figure 3:**
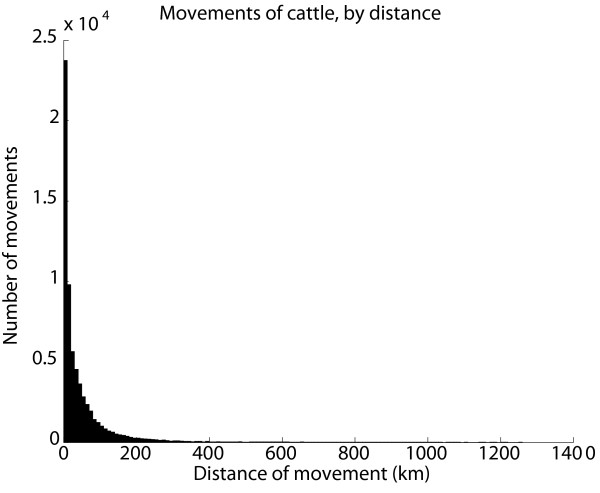
**Reported movements of cattle, by distance**. Movements of Swedish cattle during the period between July 1^st ^2005 and June 30^th ^2006 by distance between the holding of origin and the holding of destination. All animals transported between the same two holdings on the same date were regarded as one movement, movements of cattle to pasture are not included.

**Figure 4:**
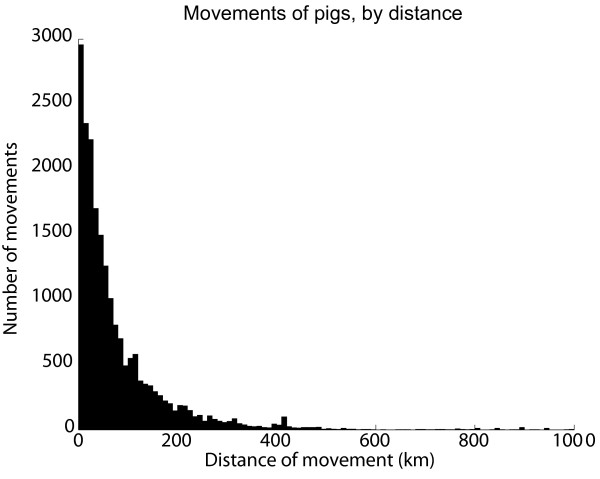
**Reported movements of pigs, by distance**. Movements of Swedish pigs during the period between July 1^st ^2005 and June 30^th ^2006 by distance between the holding of origin and the holding of destination. All animals transported between the same two holdings on the same date were regarded as one movement.

We found that most movements of livestock were within quite short distances; 87% of the cattle movements and 74% of the pig movements were to holdings within 100 km. However 5% of the cattle movements and 9% of the pig movements were to holdings more than 200 km away, with distances up to 1200 km for cattle and 1000 km for pigs. (Figure 3, Figure 4) These findings are also reflected in the analysis of movements within and between counties (Table 2). Most movements were within a county or to a nearby county, but there were also a considerable number of movements between counties that were further apart. Movements between the three larger regions are shown in Table 3. For cattle the geographical patterns of trade between counties were quite similar for different age classes of cattle, with by far the most trade in the age class below six months of age (detailed data not shown).

**Table 2:** Movements of cattle and pigs by county

**Movements starting in county**	**Cattle**			**Pigs**		
		
	**Number of movements**	**Within county** **(%)**	**To adjacent county** **(%)**	**Number of movements**	**Within county (%)**	**To adjacent county** **(%)**
Stockholm	879	48.6	36.1	70	41.4	40.0
Uppsala	2094	67.6	22.2	644	24.2	39.1
Södermanland	1722	64.7	27.8	389	10.8	62.0
Östergötland	4183	69.6	20.2	1378	67.9	20.0
Jönköping	5926	76.2	19.0	441	12.7	60.5
Kronoberg	2578	70.3	24.7	199	23.1	67.8
Kalmar	4961	69.4	20.3	816	76.3	18.1
Gotland	1914	93.3	n.a.*	621	77.0	n.a.*
Blekinge	1209	50.0	46.2	201	54.7	39.3
Skåne	7802	89.9	5.5	6902	73.2	14.4
Halland	3626	63.3	34.2	2775	65.3	29.4
Västra Götaland	11869	87.8	8.8	3562	73.8	17.8
Värmland	1825	81.1	16.9	376	70.5	22.9
Örebro	1209	75.7	18.4	445	40.0	46.3
Västmanland	988	60.9	33.2	1187	36.0	27.9
Dalarna	1557	76.6	15.7	116	35.3	20.7
Gävleborg	1697	84.3	13.9	37	86.5	10.8
Västernorrland	1282	71.8	16.3	22	81.8	18.2
Jämtland	1263	72.1	15.7	6	83.3	16.7
Västerbotten	2019	79.5	6.6	38	100.0	0
Norrbotten	866	75.2	7.9	6	33.3	66.7

Reported movements of Swedish cattle and pigs from one holding to another within county and to adjacent counties, during the period between July 1^st ^2005 and June 30^th ^2006.

All animals moved between the same two holdings the same date were regarded as one movement. Movements to pasture were not included.

*Not applicable, Gotland is an island.

**Table 3:** Reported movements of Swedish cattle and pigs from one holding to another during the period between July 1^st ^2005 and June 30^th ^2006, within and between regions

**Transports from**	**Number of transports**	**Tranports to**		
		
		**Götaland (%)**	**Svealand (%)**	**Norrland (%)**
Cattle				
Götaland	44068	97	2.8	0.2
Svealand	11971	5.1	93.2	1.7
Norrland	5430	1.3	16.8	82
Pigs				

Götaland	16895	95.1	4.3	0.6
Svealand	3264	28.9	67.7	3.3
Norrland	72	0	0	100

All animals moved between the same two holdings the same date were regarded as one movement. Movements to pasture were not included.

The long distance movements are important to consider in an acute phase of an outbreak of a disease such as FMD, that would motivate an immediate standstill of livestock transports to prevent further spread. Since movements should be reported to the databases within one week after the movement has occurred, there is a delay in the system and all recent movements cannot be found in the database. Consequently the geographical extent of a standstill could not be based only on a rapid analysis of the movements reported to the database; a decision must be based on prior knowledge of movement patterns. These data support the implementation of a total standstill in the whole country until detailed contact tracing has been done and necessary control measures have been taken.

In a later stage of an outbreak when further control measures have been implemented and the situation has stabilized, it would probably be possible to redirect long distance movements to enable regionalisation. The current basis for regionalisation in contingency plans is county level and this seems appropriate for some counties but not all; in several cases there are extensive movements to adjacent counties and in those cases it might be preferable to create regions including neighbouring counties. Moreover, other types of contacts, such as movements to slaughter and other direct and indirect contacts also need to be considered in any decision of this kind.

An estimate of the frequency of movements between holdings and the geographical patterns of these movements can be used as input for models of disease spread. The data have also been analysed to describe the distance dependence of movements mathematically using spatial Kernel functions. It was shown that the transports were preferably modelled as a mixture of distance dependent and distance independent processes [28].

The movement patterns identified might partly explain the regional clustering of some endemic diseases within Sweden, such as a certain clone of VTEC in the county of Halland [29] and Salmonella Dublin on the island of Öland. The knowledge could be useful when designing or revising existing control programs, e.g. identifying movement patterns from known high prevalence areas to areas with lower prevalence and using the information to target sampling.

**Figure 5:**
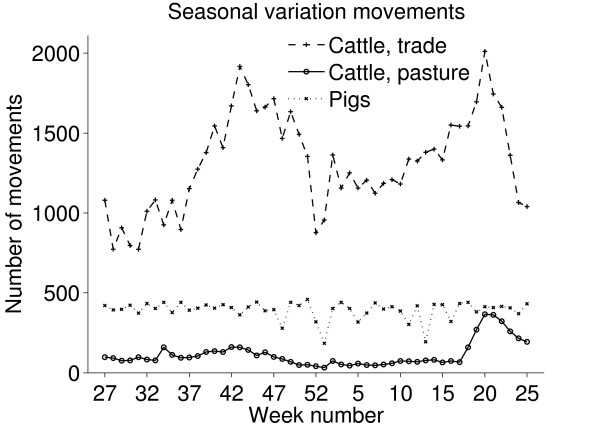
**Reported movements of cattle and pigs, seasonal variation**. Number of reported movements of Swedish cattle and pigs from one holding to another per week during the period between July 1^st ^2005 and June 30^th ^2006. All animals transported between the same two holdings on the same date were regarded as one movement. (The first week included only 5 days, and the last week only two days and therefore not shown in the graph.)

**Figure 6:**
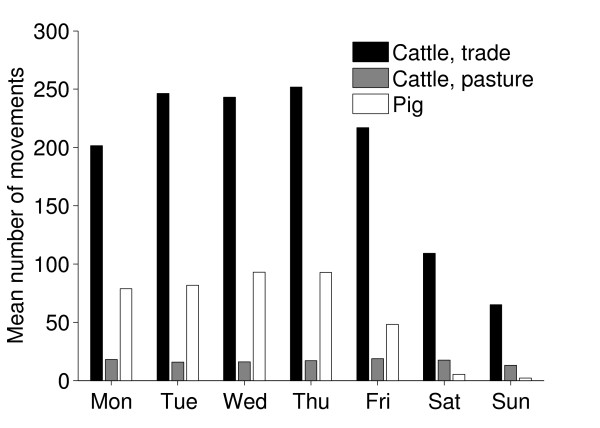
**Reported movements of cattle and pigs per day of the week**. Mean numbers of reported movements of Swedish cattle and pigs from one holding to another per day of the week (national holidays were treated as Sundays) during the period between July 1^st ^2005 and June 30^th ^2006. All animals transported between the same two holdings on the same date were regarded as one movement.

Results of the temporal investigations of the movements are shown in Figures 5 and 6. Notably there was a seasonal variation in the movements of cattle with peaks in movements during May and in October and November, similar patterns have been observed in the UK [17]. For both cattle and pigs there was a clear dip in the number of movements during Christmas holidays (Figure 5). Most movements occurred on weekdays, except for movements of cattle to pasture which were quite evenly distributed throughout the week (Figure 6). The figures do not give a true picture of the total number of movements to pasture, since only movements to pasture which are not part of the holding where the animals are kept are reported. However we believe these data reflect the temporal patterns of movements to pasture, and there is a clear peak in the movements to pasture in springtime. A similar peak is not seen in autumn, the reasons might be that the return of animals from pasture is a more prolonged or that animals are sent directly to slaughter from pasture (Figure 5).

### Trade

**Figure 7:**
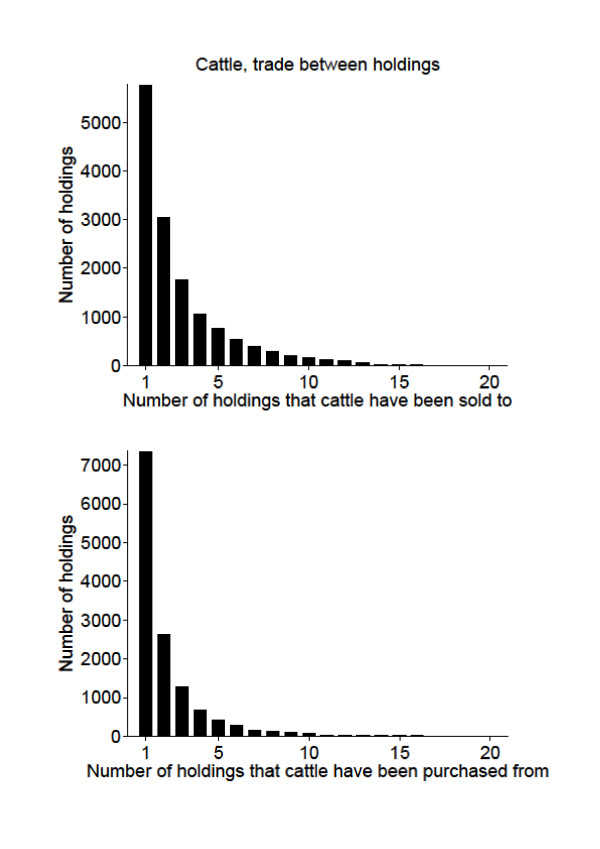
**Trade between holdings, cattle**. Trade between Swedish cattle holdings during the period between July 1^st ^2005 and June 30^th ^2006, shown by the number of holdings that cattle have been sold to or purchased from. There were 15502 cattle holdings that did not sell cattle and 16366 that did not purchase cattle during the period. There were 47 holdings that sold to more than 20 holdings and 252 that bought from more than 20 holdings.

**Figure 8:**
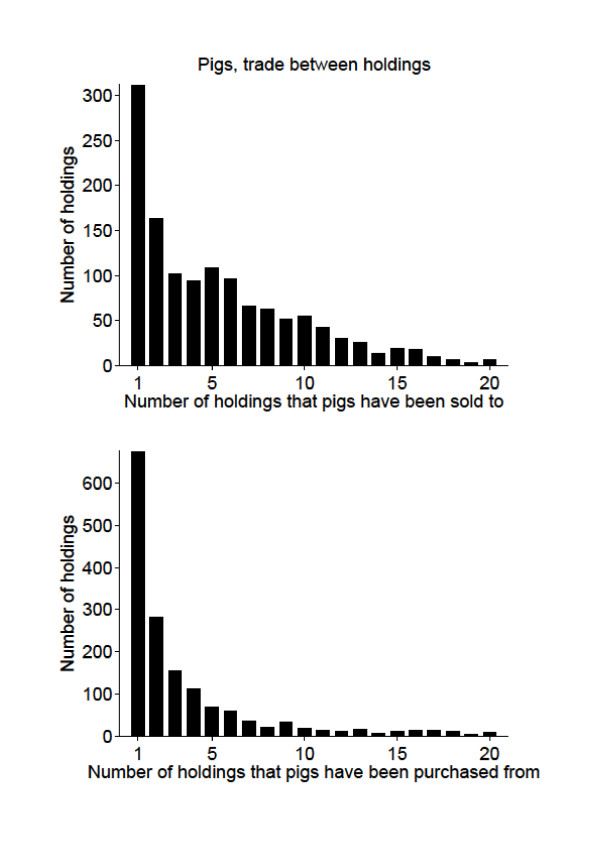
**Trade between holdings, pigs**. Trade between Swedish pig holdings during the period between July 1^st ^2005 and June 30^th ^2006, shown by the number of holdings that pigs have been sold to or purchased from. There were 1787 pig holdings that did not sell pigs and 1421 that did not purchase pigs during the period. There were 63 holdings that sold to more than 20 holdings and 115 that bought from more than 20 holdings.

Holdings with extensive trade can be important for disease control and surveillance; the ones selling to many holdings could spread the disease effectively in case of an outbreak or endemic disease. Likewise, holdings buying animals from many holdings will have increased risk of introduction. Thus, such holdings are important to target for investigations and information during an outbreak. The trade patterns of holdings are shown in Figures 7 and 8. Out of 30407 cattle holdings; 51% of the holdings did not sell cattle to other holdings, and 54% of the holdings did not purchase any cattle. For the 3165 pig holdings, 56% of the holdings did not sell pigs to other holdings, and 45% of them did not purchase any pigs.

The maximum number of holdings that a cattle holding sold cattle to was 85, and the maximum number of holdings a pig holding sold pigs to was 106. The maximum number of holdings cattle were purchased from to one holding was 392 and finally, the maximum number of holdings that pigs were purchased from to one holding was 107. It would have been interesting to perform further analyses on these holdings related to their type of production, especially for the cattle holdings. But the cattle data did not contain information on production type, and therefore further analyses could not be done. As already mentioned, analyses related to age were done and most cattle traded were calves. Many of the cattle movements could be calves from dairy herds being sold to fattening herds, however this cannot be analysed from the available data.

### Geographical distribution of holdings

**Figure 9:**
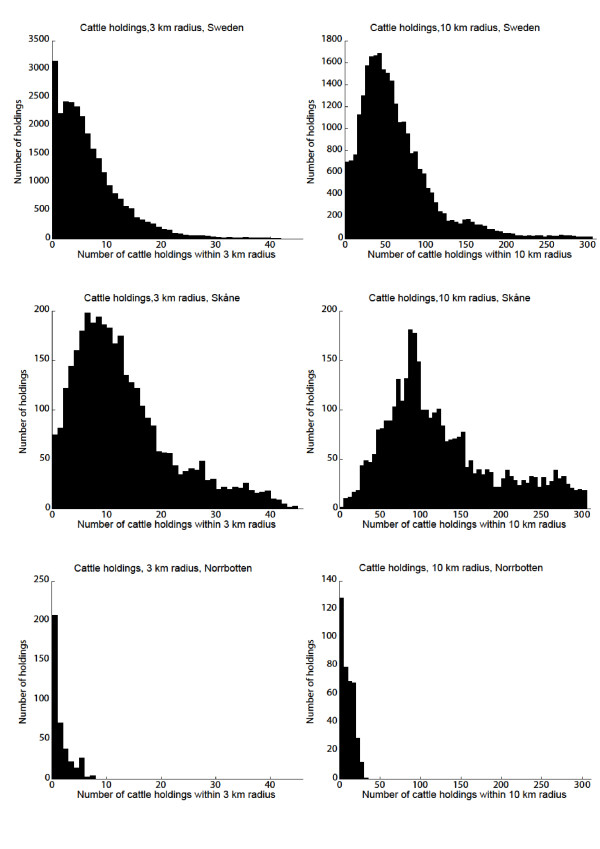
**Cattle holdings within 3 and 10 km**. Number of registered, active cattle holdings within 3 and 10 km radius from each cattle holding, in Sweden, Norrbotten and Skåne.

**Figure 10:**
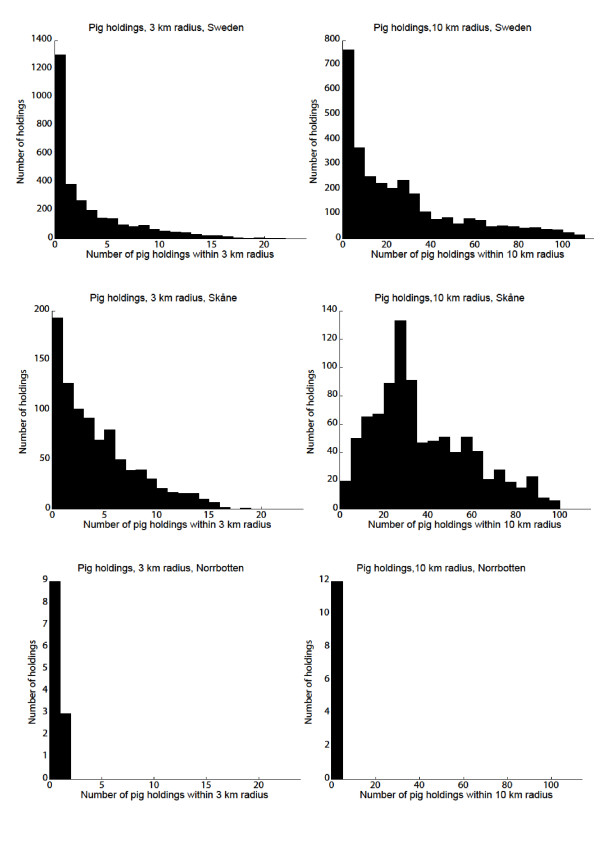
**Pig holdings within 3 and 10 km radius**. Number of registered, active pig holdings within 3 and 10 km radius from each pig holding, in Sweden, Norrbotten and Skåne.

The geographical distribution of the holdings is shown in Figures 1 and 2, and the number of holdings within 3 km and 10 km radius from each holding are shown in Figures 9 and 10. The maximum number of cattle holdings within a 3 km radius from one cattle holding was 45. The maximum number of pig holdings within a 3 km distance from a pig holding was 23. There were large variations among counties with a clear south-north gradient and as illustrative examples two extremes (Skåne and Norrbotten) are shown in Figures 9 and 10. In the northern parts of the country many holdings did not have any other holdings even within the 10 km radius. These analyses gave us a more detailed insight in the geographical distribution of holdings in relation to protection and surveillance zones that might be implemented in case of a future outbreak.

### Births and deaths

**Figure 11:**
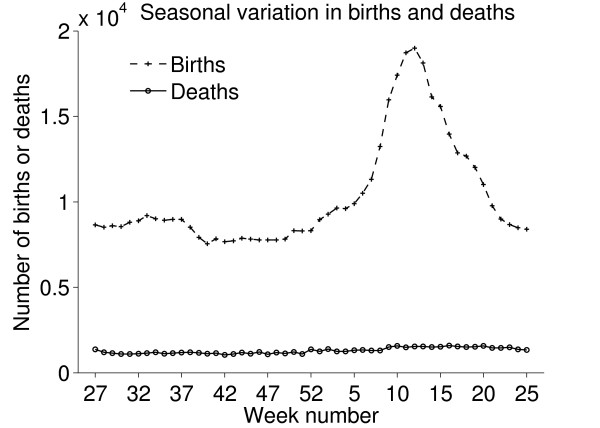
**Reported births and deaths, seasonal variation**. Number of reported births and deaths of Swedish cattle per week during the period between July 1^st ^2005 and June 30^th ^2006.

**Figure 12:**
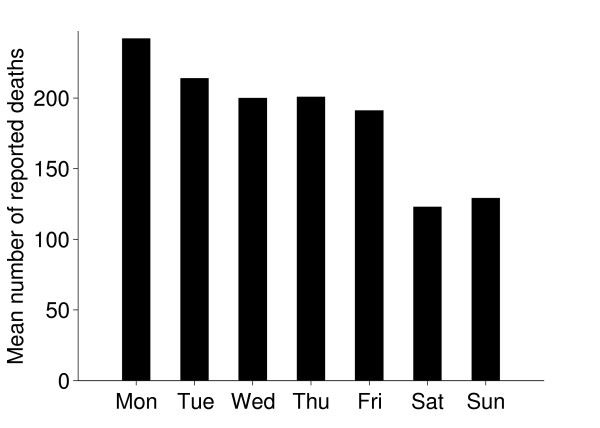
**Reported deaths per day of the week**. Mean numbers of reported deaths of Swedish cattle per day of the week, during the period between July 1^st ^2005 and June 30^th ^2006.

**Figure 13:**
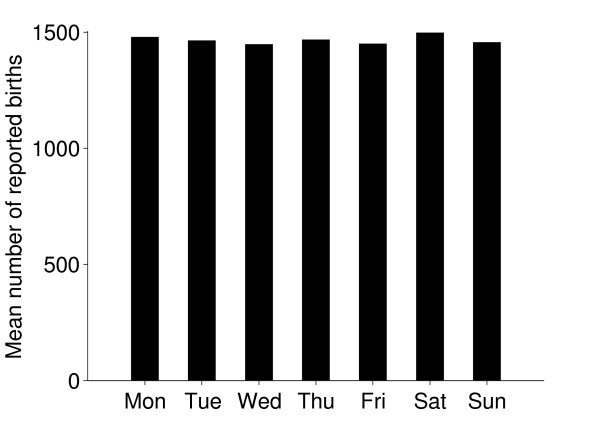
**Reported births per day of the week**. Mean numbers of reported births of Swedish cattle per day of the week, during the period between July 1^st ^2005 and June 30^th ^2006.

**Figure 14:**
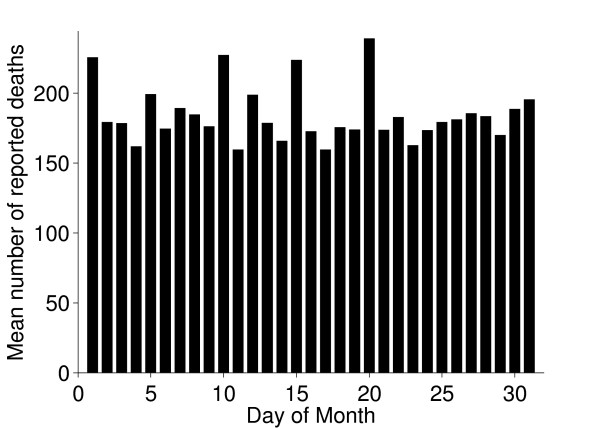
**Reported deaths per day of the month**. Mean numbers of reported deaths of Swedish cattle per day of the month, during the period between July 1^st ^2005 and June 30^th ^2006.

**Figure 15:**
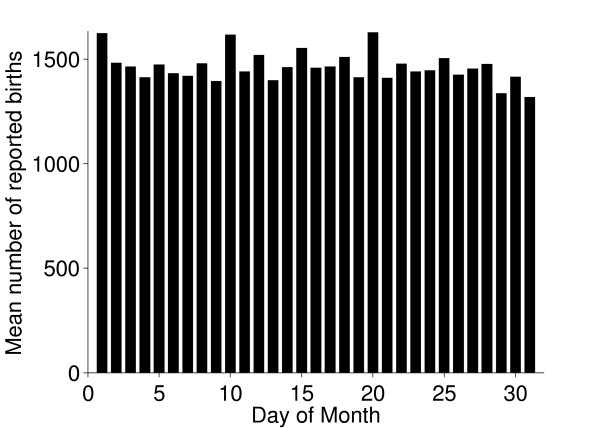
**Reported births per day of the month**. Mean numbers of reported births of Swedish cattle per day of the month, during the period between July 1^st ^2005 and June 30^th ^2006.

The seasonal patterns of cattle births were much as expected; most beef cattle have their calves in spring. There were also more deaths during spring and this might be a consequence of disorders and subsequent culling related to calving (Figure 11). Reported births and deaths were not evenly distributed among the days of the week (chi-square test, p < 0.001). Deaths were reported far more often on weekdays, especially Mondays and Tuesdays, compared to weekends. (Figures 12 and 13) We believe that farmers might be reluctant to call the veterinarian or the knacker Saturdays and Sundays due to higher fees during weekends, which explains these results. Reported births and deaths were not evenly distributed among the days of the month (chi-square test, p < 0.001) (Figure 14 and 15). We found that more deaths than expected were reported on the 1^st^, 10^th^, 15^th ^and 20^th^. In the seven different tests (one for each day of the week) the reported number of deaths on these dates was never below expected and up to 48% more than expected, with a median of approximately 20% more than expected. Although the reported births deviated less from the expected compared to reported deaths, approximately 10% more births than expected were reported the 1^st^, 10^th ^and 20^th^. We believe this is due to recall bias in combination with digit preference, which is also reported to occur in the UK cattle database [17]. The differences between reported births and deaths indicate that farmers were more accurate when reporting births compared to reporting deaths.

### Bias and data quality

In addition to the digit preference found in the reporting of death dates, inconsistencies were found in the cattle movement data. We identified that 20% of the reports of cattle traded did not have a corresponding report, which they normally should. The details of these inconsistencies are shown in Table 1. The major discrepancy was due to different dates in the reports, which represented 70% of the non matching reports. There is a possibility that reporting has been correct even though there are different dates in the reports; the animal might arrive on the holding of destination the day after leaving the holding of origin but this would not explain all the non matching dates. After data editing 0.2% of the reported movements could not be included in the analysis, this was due to inconsistent routes of movement or animals not reported to be on the same holding at the end of the period as they would have been if the reported transports had been correct.

Since movements of pigs are single reports by the holding that receive the animals, and births and deaths are not reported, we could not assess the quality of the reported pig movements but we assume there might be missing or incorrectly reported pig movements in the database.

Our choice to include only active holdings might have underestimated the true number of holdings. However, including all holdings in the register would overestimate the number of holdings, since there are inactive holdings in the database. A comparison with the yearly agricultural statistics was not relevant, since it displays the number of agricultural companies and one company can have more than one holding.

Moreover, 174 holding numbers, which were present in animal movement data or data on animals present at the holding, were not present in the data from the holding database and were therefore excluded from all geographical and movements analyses. Out of these, 8 pig holdings and 68 cattle holdings were reported to have been involved in movements between holdings and as a consequence 237 reported pig movements and 143 reported cattle movements were excluded. These might be typing errors by farmers, reporting or errors in some other stage of data entry, alternatively all holdings were not included in the extract we received from the holding database. Furthermore, in the geographical analyses of holdings we excluded 73 (2,3%) of the active pig holdings and 3309 (11%) of the active cattle holdings due to missing coordinate data, and thus our results underestimate the true number of holdings within the 3 km and 10 km radius and the number of movements at certain distances and between counties.

Keeping in mind that the coverage is not complete and that erroneous reports exist in the databases, we still find the information in the databases very useful for identifying patterns and for contact tracing. The problems identified can be expected in large databases and we do not believe that these results are exceptional in any way, similar problems have also been reported from other countries [16,17]. In order to improve data quality some countries either encourage or punish farmers, e.g. by not compensating the farmer financially when the herd is depopulated due to an outbreak if the farmer has not reported movements correctly [30]. In Sweden farmers previously had to pay for each report, which was clearly not encouraging them to report. However, since 2008 the databases have been financed by the government and it would be interesting to investigate if this has had a positive effect on reporting. In addition to this, we have identified other possible improvements of the databases. We believe that quality control of reported movements and events, inclusion of geographic location of cattle farms as well as type of production for cattle holdings would further improve the usefulness of the data. Furthermore, it is important to continuously update the database to deactivate holdings where animals are no longer kept.

### Future research

The agricultural sector is in constant change and it would be interesting to investigate data from several years and analyse if there are any trends, as has been done in the UK [16,17]. It would also be interesting to analyse the movements from a network perspective, and compare to results from network analyses performed in other countries [14,15,18,31-33]. The movements to slaughter were not included within the scope of this study. However movements to slaughter can be important for disease spread; and in case of regionalisation during an outbreak the location of slaughterhouses and related transports are important and thus it would be interesting to investigate these movements.

## Conclusion

In the acute phase of an outbreak, the pig and cattle movement databases can be valuable tools in contact tracing, but cannot replace manual contact tracing using thorough interviews with farmers and hauliers, given the delays in reporting and other quality issues. Even though most movements were within a 200 km radius, some animals were moved long distances. Hence, these patterns of animal movements support an initial total standstill including the whole and not only parts of the country in case of an outbreak of FMD since the disease may already be widespread in the country at the point of detection. The analysis of the geographic location of the holdings gave valuable information for contingency planning, especially related to resource allocation.

Data on population dynamics and livestock movements are very useful and the databases contain valuable information. The problems with data quality are to be expected in databases like these, but improvement in data quality would increase the usefulness of the data even further.

## Competing interests

The authors declare that they have no competing interests.

## Authors' contributions

MN and SSL designed the project and obtained the data from the Swedish Board of Agriculture. MN, NH and TL decided in detail how to edit data and which analyses to perform. NL and TL carried out the analyses. UW and SSL supervised the project. MN drafted the manuscript and all authors contributed to the writing.

## References

[B1] Gilbert M, Mitchell A, Bourn D, Mawdsley J, Clifton-Hadley R, Wint W (2005). Cattle movements and bovine tuberculosis in Great Britain. Nature.

[B2] Green DM, Kiss IZ, Kao RR (2006). Modelling the initial spread of foot-and-mouth disease through animal movements. Proc Biol Sci.

[B3] Anonymous (2003). Council Directive 2003/85/EC of 29 September 2003 on Community measures for the control of foot-and-mouth disease. Official Journal.

[B4] Thompson D, Muriel P, Russell D, Osborne P, Bromley A, Rowland M, Creigh-Tyte S, Brown C (2002). Economic costs of the foot and mouth disease outbreak in the United Kingdom in 2001. Rev Sci Tech.

[B5] Anonymous (2001). Council Directive 2001/89/EC of 23 October 2001 on Community measures for the control of classical swine fever. Official Journal.

[B6] Brouwer-Middelesch H, Backer JA, Swart WAJM, van Roermund HJW, Wolf V, van Schaik G (2008). Development of an early warning system (EWS) for detection of classical swine fever (CSF). Society for Veterinary Epidemomiology and Preventive Medicine 2008; Liverpool, UK.

[B7] Schley D (2007). Enhancing confidence in epidemiological models of foot-and-mouth disease, in Report of the thirty-seventh session of the European Commission for the control of foot-and-mouth disease. Rome.

[B8] Tildesley MJ, Keeling MJ (2008). Modelling foot-and-mouth disease: a comparison between the UK and Denmark. Prev Vet Med.

[B9] Robinson SE, Christley RM (2007). Exploring the role of auction markets in cattle movements within Great Britain. Prev Vet Med.

[B10] Mansley LM, Dunlop PJ, Whiteside SM, Smith RG (2003). Early dissemination of foot-and-mouth disease virus through sheep marketing in February 2001. Vet Rec.

[B11] Anonymous (2000). Regulation (EC) No 1760/2000 of the European Parliament and of the Council of 17 July 2000 establishing a system for the identification and registration of bovine animals. Official Journal.

[B12] Anonymous (2008). Council Directive 2008/71/EC of 15 July 2008 on the identification and registration of pigs. Official Journal.

[B13] Anonymous (2000). 2000/678/EC: Commission Decision of 23 October 2000 laying down detailed rules for registration of holdings in national databases for porcine animals. Official Journal.

[B14] Bigras-Poulin M, Barfod K, Mortensen S, Greiner M (2007). Relationship of trade patterns of the Danish swine industry animal movements network to potential disease spread. Prev Vet Med.

[B15] Bigras-Poulin M, Thompson RA, Chriel M, Mortensen S, Greiner M (2006). Network analysis of Danish cattle industry trade patterns as an evaluation of risk potential for disease spread. Prev Vet Med.

[B16] Green DM, Kao RR (2007). Data quality of the Cattle Tracing System in Great Britain. Vet Rec.

[B17] Robinson SE, Christley RM (2006). Identifying temporal variation in reported births, deaths and movements of cattle in Britain. BMC Vet Res.

[B18] Vernon MC, Keeling MJ (2009). Representing the UK's cattle herd as static and dynamic networks. Proc Biol Sci.

[B19] Ribbens S, Dewulf J, Koenen F, Mintiens K, de Kruif A, Maes D (2009). Type and frequency of contacts between Belgian pig herds. Prev Vet Med.

[B20] Caporale V, Giovannini A, Di Francesco C, Calistri P (2001). Importance of the traceability of animals and animal products in epidemiology. Rev Sci Tech.

[B21] Yearbook of agricultural statistics 2008 including food statistics. http://www.jordbruksverket.se/download/18.50cb902d1234ca17a7e8000517/JS%C3%85+2008+Hela.pdf.

[B22] Yearbook of agricultural statistics 2007 including food statistics. http://www.jordbruksverket.se/download/18.50cb902d1234ca17a7e8000555/statistisk+%C3%A5rsbok+2007.pdf.

[B23] STUDS slutrapport Delprojekt Övervakning och Smittspårning. http://www.sjv.se/download/18.7502f61001ea08a0c7fff57496/%C3%96S_slutrapport.PDF.

[B24] Rothman KJ, Greenland S (1998). Modern Epidemiology, Second Edition.

[B25] Bopp M, Faeh D (2008). End-digits preference for self-reported height depends on language. BMC Public Health.

[B26] Miller CA, Anderson WL (2002). Digit Preference in Reported Harvest Among Illinois Waterfowl Hunters. Human Dimensions of Wildlife: An International Journal.

[B27] Broad J, Wells S, Marshall R, Jackson R (2007). Zero end-digit preference in recorded blood pressure and its impact on classification of patients for pharmacologic management in primary care - PREDICT-CVD-6. Br J Gen Pract.

[B28] Lindstrom T, Sisson SA, Noremark M, Jonsson A, Wennergren U (2009). Estimation of distance related probability of animal movements between holdings and implications for disease spread modeling. Prev Vet Med.

[B29] Kistemann T, Zimmer S, Vagsholm I, Andersson Y (2004). GIS-supported investigation of human EHEC and cattle VTEC O157 infections in Sweden: geographical distribution, spatial variation and possible risk factors. Epidemiol Infect.

[B30] Horst HS, Meuwissen MP, Smak JA, Meijs CC Van der (1999). The involvement of the agriculture industry and government in animal disease emergencies and the funding of compensation in western Europe. Rev Sci Tech.

[B31] Ortiz-Pelaez A, Pfeiffer DU, Soares-Magalhaes RJ, Guitian FJ (2006). Use of social network analysis to characterize the pattern of animal movements in the initial phases of the 2001 foot and mouth disease (FMD) epidemic in the UK. Prev Vet Med.

[B32] Robinson SE, Everett MG, Christley RM (2007). Recent network evolution increases the potential for large epidemics in the British cattle population. J R Soc Interface.

[B33] Heath MF, Vernon MC, Webb CR (2008). Construction of networks with intrinsic temporal structure from UK cattle movement data. BMC Vet Res.

